# The effects of facilitatory and inhibitory kinesiotaping of Vastus Medialis on the activation and fatigue of superficial quadriceps muscles

**DOI:** 10.1038/s41598-022-17849-x

**Published:** 2022-08-04

**Authors:** Peyman Aghaie Ataabadi, Ali Abbasi, Mohsen Shojaatian, Amir Letafatkar, Zdenek Svoboda, Giacomo Rossettini

**Affiliations:** 1 Department of Biomechanics and Sports Injuries, Faculty of Physical Education and Sports Sciences, Kharazmi University, Tehran, Iran; 2 Department of Sports Biomechanics and Motor Control, Faculty of Physical Education and Sports Sciences, Mazandaran University, Babolsar, Iran; 3 Faculty of Physical Culture, Palacký University Olomouc, Olomouc, Czech Republic; 4 School of Physiotherapy, University of Verona, Via Bengasi 4, 37134 Verona, Italy; 5 Biomechanics and Corrective Exercise Laboratory, Faculty of Physical Education and Sport Sciences, Kharazmi University, Mirdamad Blvd., Hesari St, Tehran, Iran

**Keywords:** Physiology, Health care

## Abstract

This study aimed to investigate how facilitatory and inhibitory KT of the Vastus Medialis affected the activation and the fatigue indices of VM, Vastus Lateralis (VL) and Rectus Femoris (RF) throughout a dynamic fatigue protocol. Seventeen collegiate athletes (Ten males, seven females, age: 24.76 ± 3.99 years, height: 1.73 ± 0.10 m, mass: 68.11 ± 8.54 kg) voluntarily participated in four dynamic fatigue protocol sessions in which no-tape (control condition), inhibitory, facilitatory and sham KTs were applied to the Vastus Medialis in each session. The protocol included 100 dynamic maximum concentric knee extensions at 90°/s using an isokinetic dynamometry device. The knee extensor muscle activities were recorded using wireless surface electromyography. The average muscle activity (Root mean square) during the first three repetitions and the repetitions number of 51–100, respectively, were used to calculate the before and after exhaustion muscle activity. Furthermore, median frequency slope during all repetitions was reported as the fatigue rate of muscles during different KT conditions and for the control condition (no-tape). The results showed neither muscle activation (significance for the main effect of KT; VM = 0.82, VL = 0.72, RF = 0.19) nor fatigue rate (significance for the main effect of KT; VM = 0.11 VL = 0.71, RF = 0.53) of the superficial knee extensor muscles were affected in all four conditions. These findings suggest that the direction of KT cannot reduce, enhance muscle activity or cause changes in muscle exhaustion. Future studies should investigate the generalizability of current findings to other populations.

## Introduction

Kinesiotaping (KT) is commonly used among coaches, clinicians and athletes to preserve soft tissues and joints without functional limitations^1–3^. In addition, KT has been reported to be helpful in the development of joint mobility^4,5^, resulting in an earlier occurrence of muscle peak torque^6^, improved functional performance^7^, pain relief^8^, and increased blood circulation^9^. Another claimed property of KT, given its application direction, is the modulation of muscle activity by two inhibitory and facilitatory techniques. Thus, the application of KT from insertion to origin is postulated to inhibit muscle activity by stretching the Golgi tendon organ. In contrast, applying KT from origin to insertion can facilitate muscle activity by stimulating the muscle spindle^10,11^. Accordingly, the proposed inhibitory and facilitatory techniques were also suggested as a treatment strategy for different musculoskeletal disorders, such as muscle imbalances^12^, patellofemoral pain syndrome^8^, shoulder impingement syndrome^13^, lateral epicondylitis^14^ and ankle instability^15^.

Despite facilitatory and inhibitory KT methods being progressively used in injury prevention and rehabilitation programs among athletes^16^; previous experiments reported inconsistent findings of the effects of KT on muscle activity. Some studies supported the impact of these techniques on muscle activity^17^ and on various factors such as muscle fatigue^18,19^, muscle contraction timing^20^ and muscle strength^8^. In contrast, other evidence rejected the ability of KT to influence muscle activity^17,19,21–23^. Nevertheless, these studies have not addressed the effect of KT on muscular activation when the muscles are fatigued. Indeed, the effects of KT on muscle activity may differ between normal and fatigue conditions because electromyography (EMG) amplitude and muscular activity increase during the fatigue process^24^. On the other hand, if these KT techniques could enhance or reduce muscular activity, they might either delay or accelerate muscle fatigue in the long run^25^. Consequently, it allows us to examine how facilitatory and inhibitory KT affect muscle activation. Nonetheless, limited knowledge is available on this issue^18,26^, and they are not systematically suited to identify muscular exhaustion specifically for the muscle with KT. They have reported that inhibitory KT can reduce or delay muscular fatigue during a fatigue protocol, but this index is based on torque changes at the beginning and end of the fatigue protocol^18,26^. Since they did not consider the involvement of the agonist muscles in generating the torque, it needs to separately measure the fatigue rate for each muscle using another technique as the slope of median frequency, based on time–frequency domain analysis^27,28^.

The EMG signal informs on the muscle fatigue process as they can assess muscle fibre recruitment and the conduction velocity of the electric signal via the excitable membranes^29,30^. A negative slope of median frequency (MDF), based on the Time–frequency processing method of EMG signals, is considered an indicator of fatigue onset^27,28^. Therefore, monitoring this variable during repetitive tasks aimed at detecting muscle fatigue after applying KT to the desired muscle might be helpful. Furthermore, the slope of MDF provides a specific fatigue index for each muscle, so it could be possible to evaluate the behaviour of non-taped muscles that are agonistic to taped muscles. For example, exhaustion or over-activity of one quadriceps femoris muscle has been shown to affect the activation and recruitment of other muscles in this group^31^; as a result, when one of the quadriceps femoris muscles becomes tired, the load distribution throughout the whole quadriceps femoris alters^32^. Then, if the KT techniques can affect muscular fatigue and activity in taped muscles, it should also affect other quadriceps femoris muscles. Hence, analysing the performance of un-taped muscles can offer further information regarding the effect of KT on muscle activity.

This study aimed to evaluate the effects of inhibitory and facilitatory KT of the Vastus Medialis (VM) on the activation and fatigue process of VM, Rectus Femoris (RF), and Vastus Lateralis (VL). It was hypothesized that: (1) inhibitory and facilitatory KT have effects on superficial quadriceps muscle activity pre- and post-fatigue; and (2) inhibitory and facilitatory KT affects superficial quadriceps muscle fatigue. The VM was chosen for KT because it is widely used in treating patellofemoral pain syndrome^8,15,20^ and can monitor the activation of its agonist muscles using superficial EMG.

## Methods

### Participants

Seventeen physically active collegiate students (10 males, 7 females, age: 24.76 ± 3.99 years, height: 1.73 ± 0.10 m, body mass: 68.11 ± 8.54 kg) voluntarily participated in this study. They reported no history of surgery or musculoskeletal injuries such as muscle or ligament rupture, joint laxity, or bone fracture within the previous twelve months. Before participating, all subjects were briefed about the objectives and provided written informed consent, and all participants provided written informed consent prior to enrolment. This study was performed following the Helsinki declaration^33^, its later amendments and local ethics committee by the Research Ethics Committee of the Sport Science Research Institute (IR.SSRI.REC.1400.1010).

### Instrumentation

An isokinetic dynamometer device (Biodex System 3, New York, USA) was used to measure the isokinetic concentric torque of the knee extensor. The EMG activity simultaneously was collected using 8-channel electromyography wireless (DataLITE, Biometrics Ltd, Gwent, UK) with a sampling rate of 2000 Hz during testing^34^. Bipolar active electrodes with a fixed centre-to-centre inter-electrode distance of 20 mm were used to record EMG signals.

### Data collection

**Figure 1:**
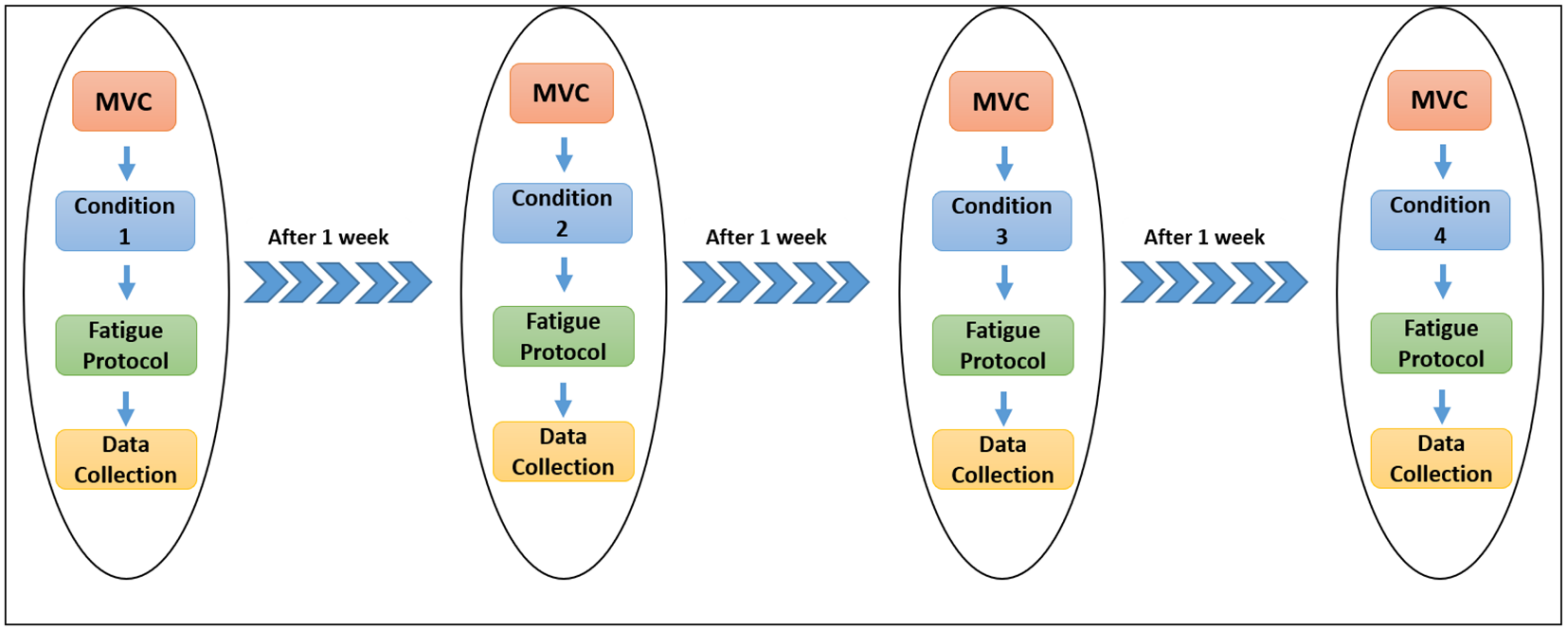
Flow diagram for the study. Conditions 1 to 4 are included no-tape, sham, facilitatory and inhibitory in a random order for each subject.

The measurements were obtained throughout four sessions (Fig. 1), with a one-week rest interval between each session. Each measurement session included five sequential phases:

1. *Warm Up* Before evaluating the measurements, all participants performed a general warm-up consisting of 5 min cycling (Biodex System 3 cycling ergometer) at 70 revolutions per minute (RPM) and 10 min of dynamic and static stretching for lower extremity muscles^35^.
2. *Electrode attachment* The electrodes were placed on the RF, VM and VL muscles of the dominant leg (self-reported ball-kicking leg test^36^), which was carefully shaved and cleaned using isopropyl alcohol by a trained researcher (Fig. 1). The active bipolar electrodes were placed on the bulky part of the selected muscles according to the guidelines of the SENIAM^37^ by the same examiner. Larson et al. show that despite the potential for error in identifying the electrode placement in four testing sessions, monitoring RMS and MNF values throughout several sessions (one week gap) has adequate reliability^38^.
3. *Maximum Voluntary Contraction Test* Then, three maximum voluntary isometric contractions at 75° flexed knees while a 2-min rest period was established between trials^39^. Participants sat on the isokinetic dynamometry seat, and the backrest and attachments on the dominant leg were set according to the manufacturer guidelines.
4. *KT applications* In each session, after the MVC test, one of the four KT modes was randomly implied on VM, including: No-tape (control group); sham KT; facilitatory KT; and inhibitory KT (one-week rest interval between each session). Then after, a 15-min rest is performed to eliminate the fatigue effects of the MVC test and improve the KT efficiency^40^.
5. *Fatigue protocol* After familiarization at the submaximal level, all subjects performed 100 dynamic maximal concentric knee extensions at a constant velocity of 90°/s while the torque and joint angle were measured using a dynamometer; muscles activity was also recorded during this exercise.

### Taping protocol

**Figure 2:**
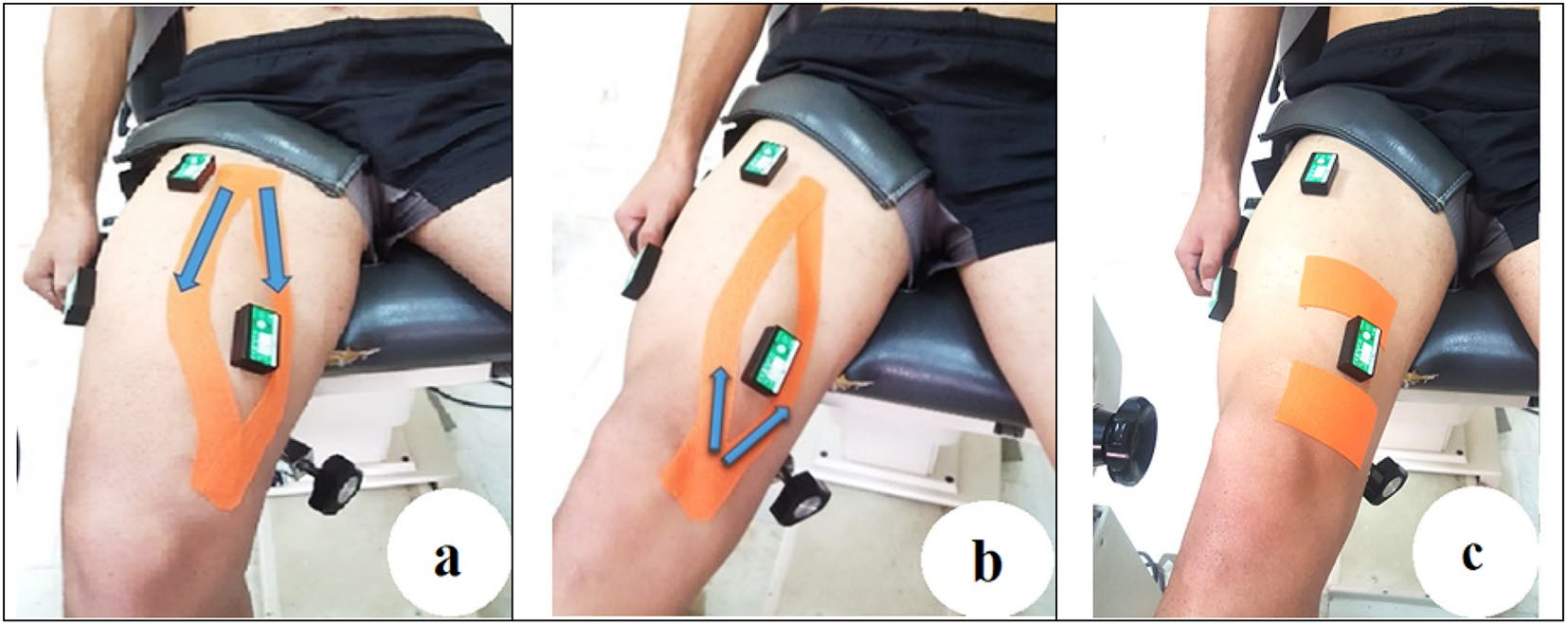
Illustration of the taping techniques. (**a**) facilitating, (**b**) inhibitory, (**c**) sham Kinesio tapings of the Vastus Medialis muscle group.

A trained researcher performed three KT on VM: sham, inhibitory and facilitatory. Each taping was randomly applied in a separate session [www.randomization.com]^23^. The tape was a waterproof KT (Ares, Korea, 5 cm wide and 0.05 cm thick). For the sham tape, two pieces of 15 cm × 5 cm tapes were applied horizontally above and below/under the muscle bulk (Fig. 2)^26^. For the inhibitory taping, the KT was applied from the insertion to the origin, while the opposite direction was used in the facilitatory taping^8,41^. In both facilitatory and inhibitory modes, the percentage of tension was 50% its available (tape was first stretched to its 100% available tension and was marked by the ruler), and in the sham mode, there was no tension^42^.

### Fatigue protocol

The participants sat in a dynamometer chair with their spine and femur aligned at a 110-degree angle. The participants practised at a submaximal contraction level to become accustomed to the equipment after placing the electrodes and warming up, and then they rested for a short while^38^. Next, they conducted 100 repeated isokinetic contractions utilizing the dominant leg knee extensors from 90 degrees of flexion to 0 degrees of flexion (full extension). The dynamometer was operated at a constant speed of 90°/s. While the arm of the dynamometer moved up from 90 to 0, subjects were instructed to perform maximally for each contraction across the entire range of motion (e.g., the contraction cycle's active phase). The subjects became relaxed when the dynamometer arm returned to 90 degrees (the passive phase of the contraction cycle). As a result, each contraction and relaxation period lasted one second, making two seconds for the contraction cycle. The 100 contractions were completed by all of the participants^38,43,44^.

### EMG data collection and processing

**Figure 3:**
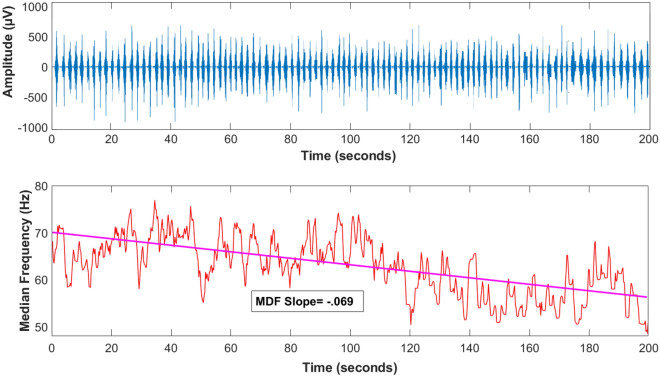
EMG Parameters. (top) Sample of electromyography activity, and (bottom) median frequency (STFT) of Rectus Femoris muscle during the fatigue protocol.

We considered root mean square (RMS) and slope of median frequency (MDF), respectively, to quantify the muscular activity and fatigue rate, as these variables had commonly been used in previous studies^24,45,46^. The raw EMG signals were filtered using a zero-lag band-pass Butterworth filter at 20 and 400 Hz cut-off frequencies. Then, the root mean square (RMS, 50-ms windows) was calculated, and the muscle activity for each repetition was determined by calculating the mean of RMS values through knee extension (active phase) was normalized to MVIC^46^. To investigate the effects of KT in both pre and post-fatigue situations, the average muscular activity was assessed in three initial extensions and 51–100 repetitions, respectively^38^; for this purpose, each cycle was separated from 90° flexion to 0° based on dynamometer angle data. Since the torque reduction in the fatigue protocol is considerably significant during the initial 40–60 contractions^43^, the average of the above repetitions has been considered as representing of pre-fatigue and post-fatigue conditions in the current research^38^. The values of the MDF were obtained using the short-time Fourier transform (STFT) technique for spectral analysis^45^. The STFT was obtained recursively (over 0.5 s time-windowed signals) using a 1024 point FFT (discrete and fast FT) with a rectangular processing window algorithm available in MATLAB® v.7.7. After applying the STFT, the MDF was obtained for further spectral analysis. The MDF, which is defined as the value of frequency that derives the EMG signal spectrum into two parts with equal energy, was calculated. Finally, the slope of the MDF was detected using linear regression between the MDF duration of the test (Fig. 3)^47^. The entire data analysis was conducted using MATLAB software (version 2020a, MathWorks, Inc., Natick, MA, USA).

### Statistics analysis

The Schapiro-Wilk test was used to check the normality of the data distribution. A two-way repeated-measures analysis of variance (ANOVA) was employed to analyse the taping condition (three types of taping), time (differences between the first and last five repetitions) and their interaction effects on the muscle activity. A one-way repeated measures ANOVA was used to compare the effects of four situations of taping (no-tape, sham, inhibitory and facilitatory) on the MDF slope of the RF, VM, and VL muscles during the fatigue protocol. The significance level was set at α = 0.05, and all statistical analyses were conducted using the SPSS statistical package (version 25, IBM Corporation, Armonk, NY, USA).

### Ethics approval and consent to participate

This study was ethically approved by the Research Ethics Committee of Sport Sciences Research Institute (Iran) according to compliance with Ethical Standards in Research of the Ministry of Science, Research and Technology (code: IR.SSRI.REC.1400.1010).

## Results

The descriptive results of the RF, VM, and VL muscles activity in the no-tape, sham, inhibitory and facilitatory conditions are reported in Table 1 and the findings of the two-way repeated-measures ANOVA are reported in Table 2. These results indicated that the main effects of KT, Time and Interaction (Time × KT) were not significant on the muscle activity of the RF (KT: F = 1.65, p = 0.19; Time: F = 0.4, p = 0.53; Interaction: F = 0.51, p = 0.67), VL (KT: F = 0.4, p = 0.72; Time: F = 2.3, p = 0.14; Interaction: F = 0.8, p = 0.96), and VM (KT: F = 0.31, p = 0.82; Time: F = 2.4, p = 0.14; Interaction: F = 1.14, p = 0.34) (Table 2).

**Table 1:** RF, VL, and VM activity (RMS in %MVC) in four different conditions (no-tape, sham, inhibitory and facilitatory KT conditions).

Muscles	Conditions	Pre_fatigue	Post_fatigue	Differences	t (2-tailed)	Sig
RF	No-tape	83.4 ± 22.1	81.1 ± 17.2	2.2 ± 21.7	0.43	0.67
	Sham	81.5 ± 25.3	83.2 ± 17.4	− 1.74 ± 19.8	− 0.36	0.72
	Inhibitory	78.5 ± 18.4	75.7 ± 17.2	2.8 ± 24.3	0.48	0.63
	Facilitatory	90.4 ± 26.1	84.3 ± 15.5	6.1 ± 21.8	1.13	0.27
VL	No-tape	88.7 ± 23.5	81.7 ± 26.3	6.9 ± 28.5	1.01	0.33
	Sham	80.6 ± 26.5	76.6 ± 22.7	4.1 ± 22.7	0.73	0.46
	Inhibitory	86.4 ± 20.4	79.6 ± 24.1	6.7 ± 30.7	0.90	0.38
	Facilitatory	89.1 ± 27.6	81.4 ± 22.3	7.6 ± 18.5	1.66	0.11
VM	No-tape	88.7 ± 25.7	84.1 ± 19.5	4.7 ± 16.8	1.15	0.26
	Sham	81.5 ± 22.1	81.8 ± 18.2	− 0.3 ± 16.11	− 0.08	0.93
	Inhibitory	88.1 ± 24.5	79.8 ± 21.3	8.1 ± 22.6	1.48	0.15
	Facilitatory	85.1 ± 27.5	77.2 ± 20.2	7.8 ± 19.1	1.69	011

*VM* Vastus Medialis, *RF* Rectus Femoris, *VL* Vastus Lateralis.

**Table 2:** Results of the two-way repeated measures ANOVA (muscle activity before and after fatigue during four different conditions).

Muscles	Conditions			Time			Interaction (Conditions × time)		
	F (df = 3)	Sig	Partial eta squared	F (df = 1)	Sig	Partial eta squared	F (df = 3)	Sig	Partial eta squared
RF	1.65	0.19	0.09	0.4	0.53	0.02	0.51	0.67	0.03
VL	0.4	0.72	0.03	2.3	0.14	0.13	0.8	0.96	0.01
VM	0.31	0.82	0.02	2.4	0.14	0.13	1.14	0.34	0.07

*VM* Vastus Medialis, *RF* Rectus Femoris, *VL* Vastus Lateralis.

The findings of one-way repeated measure ANOVA and descriptive results of the MDF slopes of the RF, VM, and VL in the no-tape, sham, inhibitory and facilitatory conditions are reported in Table 3. According to these results, KT had no significant influence on muscular fatigue rate (MDF slopes) of RF (F = 0.73, p = 0.53), VL (F = 0.45, p = 0.71) and VM (F = 2.14, p = 0.11) (Table 3).

**Table 3:** MDF slopes of the RF, VM, and VL in four conditions (no-tape, sham, inhibitory and facilitatory KT) and one way repeated measures ANOVA results.

	No-tape	Sham	Inhibitory	Facilitatory	F	df	Sig	Partial eta squared
RF	− 0.059 ± 0.031	− 0.073 ± 0.049	− 0.066 ± 0.023	− 0.066 ± 0.024	0.73	3	0.53	0.04
VL	− 0.071 ± 0.021	− 0.065 ± 0.031	− 0.064 ± 0.021	− 0.063 ± 0.029	0.45	3	0.71	0.03
VM	− 0.069 ± 0.020	− 0.063 ± 0.037	− 0.067 ± 0.032	− 0.056 ± 0.020	2.14	3	0.11	0.12

*VM* Vastus Medialis, *RF* Rectus Femoris, *VL* Vastus Lateralis.

## Discussion

This study examined the effects of no-tape, sham, facilitatory and inhibitory KT of VM on superficial quadriceps muscle activity and fatigue. For muscular activity, the KT effect, the Time effect and the Interaction effect (Time × KT) were not significant. This data suggested no significant variations in muscle activity when various KT conditions were adopted, both in pre-fatigue and post-fatigue circumstances. For muscular fatigue, different types of KT techniques did not affect the fatigue ratio (MDF slope), neither for the VM with KT nor its agonist muscles, VL and RF.

Regarding the first hypothesis, the results showed that KT techniques did not affect muscle activation in the target muscle or its agonist muscles. In line with our findings, previous studies found no significant changes in muscle activation immediately following KT^14,21^. According to the present study and existing evidence, KT techniques do not affect muscle activity^14,21^, muscular fatigue and motor neuron excitability^48,49^, suggesting that KT techniques cannot influence the related parameter of muscle activation. However, the present study and previous research^21,43,44^ have only involved healthy individuals; the effects of various methods of KT on muscular activation in patients with musculoskeletal pain have received less attention^8,14^. Another research on healthy participants found that KT had no effect on muscular activity among both regular KT users and non-users, while it positively affected strength improvement in regular users^17^. They suggested that placebo effects as an indirect mechanism could improve strength in regular users without influencing muscular activity^17^.

Based on time–frequency analyses, our findings portrayed that KT techniques had no effects on the fatigue ratio (MDF slope) for all superficial quadriceps muscles, thus rejecting our second hypothesis. We hypothesized that the KT would change muscle target fatigue and lead agonist muscles to change their activity to keep joint torque constant during a fixed task. However, the results indicated that the KT had no impact on the chosen muscle and that there is no cause for changes in the fatigue of its agonist muscles, as regards activation and fatigue rate of agonist muscles have not been influenced in the present study. To the best of our knowledge, only one study previously used the EMG fatigue index to evaluate KT effects^50^. This study inconsistently reported that KT effectively delayed Longissimus muscle fatigue (based on median frequency slope) in people with non-specific low back pain^50^. As low back pain individuals participated in their study, while the present study only recruited healthy adults, KT may have differently influenced muscular fatigue in patients. Regardless, we deliberately used a dynamic fatigue protocol in our study because it has been claimed that the KT mechanism is related to simulating muscle spindle and Golgi tendon organs^10^, and it is recognized that these proprioceptors are making feedback as responses to the changes of length and force during dynamic movements^51^. Moreover, Abubaker et al. reported that KT could significantly postpone muscular fatigue^18^. Since they measured the fatigue ratio based on joint torque changes, it is not clear which muscles are responsible for torque changes during fatigue protocol, and the KT's impacts may have been influenced by a lack of attention to the function of other muscles. The present study used EMG signals to evaluate the fatigue index separately for VM and its agonist muscles, VL and RF; however, we could not discover any evidence of KT's effects on muscular fatigue. Therefore, due to a limited number of studies and inadequate knowledge in this area, further studies are required to consider the suitable methodology, different types of muscle contraction and other muscles in the musculoskeletal system.

## Limitations

In this study, some limitations should be acknowledged. First, since the participants of this study were healthy individuals, our findings could not be generalized to other populations of patients (e.g., presenting musculoskeletal disorders and pain or neurological injuries). Second, the results related to the joint torque (which is produced by all the knee extensor muscles) are not mentioned in the current study because the purpose was to evaluate the changes in the electrical activity of each surface quadriceps femoris muscle separately following KT applications. Finally, although a validated dynamic fatigue protocol^38^ was applied, we did not directly measure using questionnaires different factors related to fatigue rate between measurements sessions, e.g., sleep quality^52^, mental fatigue^53^ and nutrition diet^54^. However, none of the subjects reported sleep deprivation, malnutrition, or mental problems before the measurement sessions.

## Conclusion

Based on the decrease of MDF (negative slope of MDF), the designed protocol resulted in overall muscular fatigue of quadriceps muscles. However, no statistical differences existed between no-tape, sham, inhibitory and facilitatory conditions on fatigue index, neither on taped muscle nor non-taped muscles as its agonist muscles. These findings provide preliminary evidence suggesting that KT may not be able to modulate muscle activity. Finally, there is limited research investigating the effects of KT on muscular fatigue, separately in all involved muscles. On the other hand, further research is required to obtain applicable information with a wide range of applications of KT in the treatment procedures.

## Data Availability

Data would be available on a reasonable request.

## Abbreviations

KT: Kinesiotaping
VL: Vastus Lateralis
RF: Rectus Femoris
EMG: Electromyography
MDF: Median frequency
VM: Vastus Medialis
RMS: Root mean square
STFT: Short-time Fourier transform
ANOVA: Analysis of variance
MVC: Maximum voluntary contraction
MVIC: Maximum voluntary isometric contraction
